# Isolation of RNA from the Murine Colonic Tissue and qRT-PCR for Inflammatory Cytokines

**DOI:** 10.21769/BioProtoc.4634

**Published:** 2023-03-20

**Authors:** Ibrahim M. Sayed, Kaili Inouye, Soumita Das, Laura Crotty Alexander

**Affiliations:** 1 Department of Pathology, University of California San Diego (UCSD), CA 92093, USA; 2 Moore’s Comprehensive Cancer Center, UCSD, CA92093, USA; 3 Pulmonary and Critical Care Section, VA San Diego Healthcare System, La Jolla, 92161, USA; 4 Division of Pulmonary, Critical Care, Sleep and Physiology, Department of Medicine, UCSD, San Diego, United States

**Keywords:** E-cigarette, Flavored JUUL, Mouse colon, RNA, Inflammation

## Abstract

E-cigarette (E-cig) inhalation affects health status by modulating inflammation profiles in several organs, including the brain, lung, heart, and colon. The effect of flavored fourth-generation pod-based E-cigs (JUUL) on murine gut inflammation is modulated by both flavor and exposure period. Exposure of mice to JUUL mango and JUUL mint for one month upregulated inflammatory cytokines, particularly TNF-α, IL-6, and Cxcl-1 (IL-8). JUUL Mango effects were more prominent than those incurred by JUUL Mint after one month of exposure. However, JUUL Mango reduced the expression of colonic inflammatory cytokines after three months of exposure. In this protocol, we detail the process of RNA isolation from the mouse colon and the use of extracted RNA in profiling the inflammatory milieu. Efficient RNA extraction from the murine colon is the most important step in the evaluation of inflammatory transcripts in the colon.

## Background

E-cigarettes (E-cigs) were first introduced to the international market in the mid-2000s as an alternative to conventional tobacco smoking (O'Loughlin et al., 2016). E-cigs produce an aerosol (commonly called vapor) upon heating and the aerosolization of vehicle solvents propylene glycol and vegetable glycerin. In addition, a large variety of flavors are added to E-cigs to add appeal to people of all kinds, including women, children, and minorities (Zhu et al., 2014).

JUUL is one of the most popular pod-based E-cig brands. They sell pods containing e-liquids in different flavors, such as mint and mango (Huang et al., 2019). The effect of chronic inhalation of the aerosols produced from these devices on health is not yet understood.

Moshensky et al. (2022) observed in vivo mouse exposures of daily JUUL aerosol inhalation with different flavors (mango and mint) for one and three months to evaluate the effects of JUUL aerosol inhalation on the function and inflammation of different organs (Moshensky et al., 2022). The authors found that the JUUL aerosol inhalation induced inflammation in the brain, gut, and heart (Moshensky et al., 2022). A recent study using a stem cell–based approach of 3D gut organoids derived from healthy individuals revealed that E-cig induced inflammation in the gut epithelium and damaged epithelial tight junctions (Sharma et al., 2021).

Extraction of RNA from the murine colon is the most important step in the relative quantification of colon transcripts. Several factors affect the quality of extracted RNA used in qRT-PCR, such as the method used in the extraction, RNA purity and concentration, and the presence of other impurities, including guanidinium isothiocyanate, phenolic compounds, ethanol, tissue DNA, and protein. The extraction procedure should be performed efficiently, since the purity of the extracted RNA affects downstream processing, such as cDNA synthesis and PCR (Toni et al., 2018). In addition, tissue DNA and protein are other contaminants to the extracted RNA. In this protocol, we describe the detailed steps of efficient isolation of RNA from the colon of mice exposed to E-cigs with minimized levels of contamination that affect downstream processing. Also, we describe the process of quantitative measurement of the transcripts of inflammatory cytokines in the extracted RNA. For complete details of this paper and additional methods, please refer to Moshensky et al. (2022).

## Materials and Reagents

**Biological materials**

Female C57BL/6 mice (6–8 weeks)

**Materials**

1. E-cigarette devices, Kanger Mini ProTank glassomizer, https://www.vaporauthority.com/products/genuine-kanger-mini-protank-3-glassomizer
2. Juul mango and mint pods, JUUL, https://www.juul.com/shop
3. Microcentrifuge tube (1.5 mL) (Fisher Scientific, catalog number: 07 200 534)
4. 50 mL centrifuge tubes (Genesee Scientific, catalog number: 28-108)
5. 15 mL centrifuge tubes (Genesee Scientific, catalog number: 28-103)
6. Fisherbrand^TM^ wood handled cotton swabs and applicators (Fisher Scientific, catalog number: 22-363-173)
7. Petri dishes, stackable (Genesee Scientific, catalog number: 32-107G)
8. MicroAmp^TM^ optical 96-well reaction plate (Thermo Fisher Scientific, catalog number: N8010560)
9. Optical adhesive covers GPLE (Thermo Fisher Scientific, catalog number: A49767).
10. BrandTech^TM^ BRAND^TM^ thin wall 0.2 mL PCR tubes with attached caps (Fisher Scientific, catalog number: 13-882-58)

**Reagents**

1. qScript cDNA SuperMix (Quanta Biosciences, catalog number: 95048)
2. 2× SYBR Green qPCR Master Mix (Bimake, catalog number: B21203)
3. Direct-zol^TM^ RNA Miniprep kit (Zymo Research, catalog number: R2053)
4. TRI reagent (Zymo Research, catalog number: R2050-1-200)
5. Gibco^TM^ PBS, pH 7.4 (Fischer Scientific, catalog number:10-010-023)
6. UltraPure^TM^ DNase/RNase-free distilled water (Invitrogen, catalog number: 10-977-015)
7. Ethanol, pure for molecular biology (Sigma-Aldrich, catalog number: E7023)
8. RNAlater (Sigma-Aldrich, catalog number: R0901-500ML)
9. DNase I treatment preparation (this enzyme is provided with Direct-zolTM RNA Miniprep kit) (see Recipes)
10. Synthesis of cDNA from RNA using qScript cDNA Synthesis kit (see Recipes)
11. qPCR reaction mixture (see Recipes)

## Equipment

1. Noyes spring scissors, angled (Fine Science Tools, catalog number: 15013-12)
2. Fine forceps (Fine Science Tools, catalog number: 11254-20)
3. Micropipette (Eppendorf, catalog number: M22873H)
4. E-cigarette InExpose system (SCIreQ)
5. Balance (METTLER TOLEDO, Balance XPR204S)
6. Fisherbrand^TM^ 150 handheld homogenizer motor (Thermo Fisher Scientific, catalog number: 15-340-168)
7. ST8 microcentrifuge (Thermo Scientific, catalog number: 75-667-200)
8. MiniAmp Plus thermal cycler (Applied Biosystems, catalog number: A37835)
9. QuantStudio^TM^ 3 real-time PCR system, 96-well, 0.2 mL (Thermo Fisher Scientific, catalog number: A28137)

## Software

1. GraphPad Prism, RRID: SCR_002798
2. QuantStudio Design & Analysis Software

## Procedure

- **Isolation of colon from the exposed mice (Figure 1)**
  1. Expose female C57BL/6 mice (6–8 weeks) to E-cig aerosols produced from JUUL mango or JUUL mint or to air for one and three months using the InExpose system.
*Note: A detailed protocol on mouse exposure and composition of JUUL pods is present in the original article (Moshensky et al., 2022). Briefly, C57BL/6 mice (6–8 weeks of age) were placed in an exposure chamber and exposed to E-cig aerosol for 20 min three times daily, for a total of 60 min per day, for 4–12 weeks. All experiments were conducted with the approval of the UCSD Institutional Animal Care and Use Committee (IACUC protocol S16021).*
  2. At the end of exposure, euthanize mice by regulated slow (~50% flow rate) carbon dioxide inhalation followed by cervical dislocation.
  3. Using scissor and forceps, cut the whole colon of a mouse from the anus to the cecum **(Figure 1)**.
    **Figure 1.**
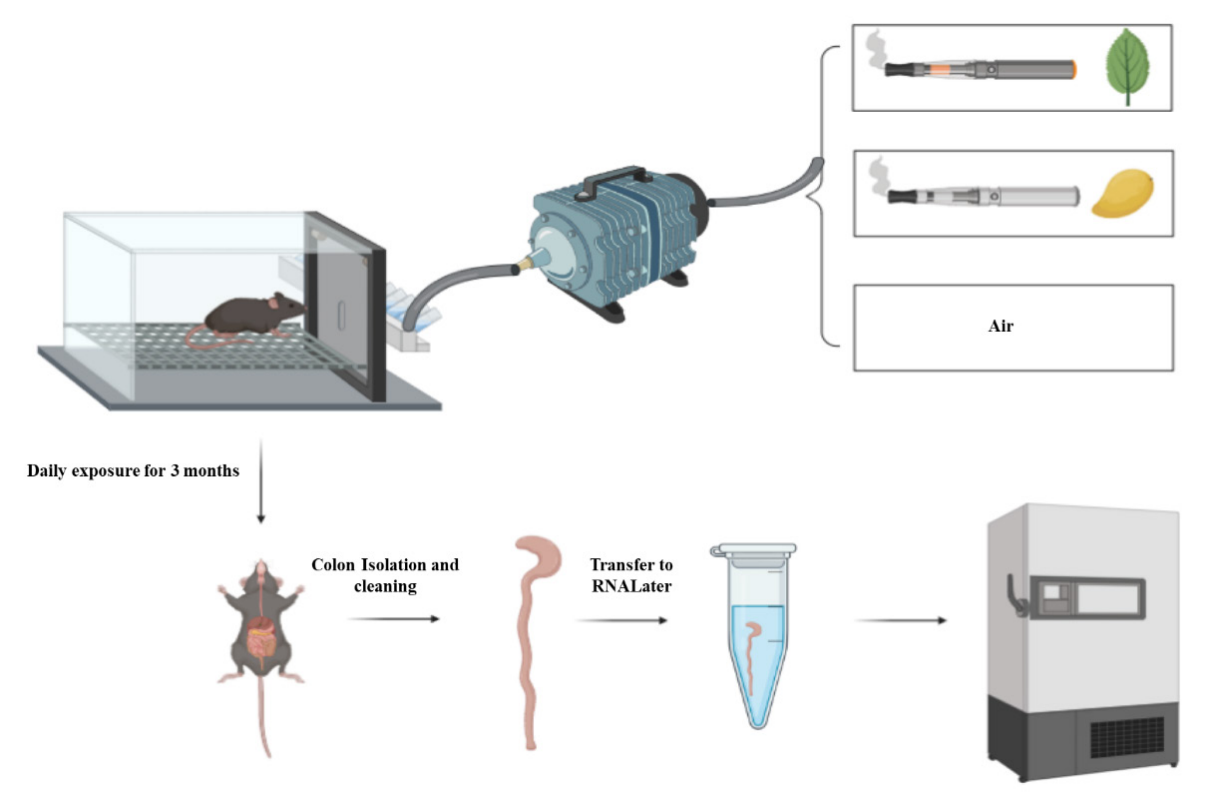
Flow of the design experiment. Mice are exposed either to air or Juul pods (mango or mint) through the InExpose system. After three months of exposure, mice are sacrificed and colons are collected, cleaned from any remaining stool, and then stored in RNAlater solution. The colon is either processed immediately or stored at -80 °C until processing.
  4. Put the colon in a Petri dish containing PBS to rinse away any blood. Remove any attached adipose tissue from the colon.
  5. Use a cotton swab to empty the colon from remaining stool.
  6. Cut the colon into small pieces and transfer part of the colon into a 1.5 mL Eppendorf containing 400–500 μL of RNAlater.
*Notes:*
    - *Saving the whole colon in RNAlater solution is an option if no other procedures/techniques are required, such as processing of the mouse colon into formalin-fixed paraffin-embedded blocks. Therefore, step A6 is optional.*
    - *In case histology is required for the colon, cut it into three parts: proximal, mid, and distal colon. The distal colon is used to assess the transcripts of inflammatory cytokines. Mid and proximal colons are used for histology and other purposes.*
    - *The amount of RNAlater can be increased (600 μL to 1 mL) if a larger part or the whole colon will be saved. The RNAlater should cover the part/whole colon saved as shown in Figure 1.*
  7. Store the colon samples at -80 °C.
*Note: If RNA extraction is done on the same day of euthanasia, step A7 is not required. Saving the tissue in RNAlater is important when the downstream processing will be performed later.*
- **Extraction of RNA from the colon tissues (Figure 2)**
  1. Thaw the colon samples on ice; then, use scissors and forceps to cut the colon tissues into small pieces.
*Note: Ensure that no precipitated salts are around the tissue. Wash the colon with PBS to remove any precipitated salts.*
  2. Weigh the colon piece using a scale/balance and ensure that the weight is ≤ 20 mg/piece.
*Notes:*
    - *It is very important to weigh the mouse colon; ideally, tissues between 5 and 20 mg give good RNA yield. Using larger tissue pieces gives a poor RNA yield due to column blockage during the RNA extraction process.*
    - *It is recommended to mince/cut the colon into small pieces after weighing to speed up the homogenization step.*
  3. Transfer the colon piece into a 1.5 mL Eppendorf containing 600 μL of TRI reagent solution.
  4. Homogenize the colon piece in TRI reagent solution using the 150 handheld homogenizer for 1–2 min until complete disruption of tissue.
*Notes:*
    - *It is important to clean and disinfect the homogenizer using 70% ethanol and water between samples to avoid cross contamination.*
    - *Make sure that the weighted piece is completely disintegrated and no visible tissue remains.*
    - *In case of tough tissue, you can increase the volume of TRI reagent to 800 μL and/or increase the homogenization time.*
    - *The homogenizer has three different speeds (low, middle, and high). It is recommended to use the middle speed, since the highest could be destructive to tissues if it is continued for a long time and the lowest could take a long period of time to disintegrate the tissue.*
  1. Centrifuge the tissue suspension at a maximum speed of 13,000 *× g* for 2 min.
  2. After the centrifugation step, you can see a precipitated debris at the bottom of the Eppendorf and a clear supernatant solution above it **(Figure 2)**. Collect the supernatant into a new 1.5 mL Eppendorf and discard the tube containing the precipitated debris.
  3. Mix the supernatant from step B6 with an equal volume (approximately 600 μL) of ethanol (95–100%).
  4. Transfer the previous mixture into a Zymo-Spin^TM^ IICR column in a collection tube (this column is a part of Direct-zol^TM^ RNA Miniprep kit).
*Note: The maximum capacity of Zymo-Spin^TM^ IICR is 700 μL and the volume of each sample is approximately 1,200 μL (600 μL TRI/tissue mix + 600 μL ethanol). Therefore, step B8 is repeated twice on the same column.*
  5. Centrifuge the mixture from step B8 at 13,000 *× g* for 1 min. Then, discard the flow through waste into 15 or 50 mL falcon tubes and discard the collection tube.
*Note: At this step, the flow through waste can be used to purify the protein from the mouse colon.*
  6. Transfer the Zymo-Spin^TM^ IICR column to a new collection tube.
  7. Add RNA wash buffer (a part of Direct-zol^TM^ RNA Miniprep kit) (400 µL/sample) to the column and centrifuge at 13,000 *× g* for 30 s to 1 min. Discard the flow through and transfer the column to a new collection tube.
  8. Add 80 µL of DNase I treatment preparation (**Recipe 1**) per column. Incubate the mixture with the column at room temperature for 15 min.
*Notes:*
    - *DNase I (part of Direct-zol^TM^ RNA Miniprep kit) is provided as lyophilized powder. Reconstitute it using with DNase/RNase-free water according to the manufacturer’s instructions. Mix well and then store the aliquots at -20 °C until use.*
    - *This step is highly recommended to get rid of DNA present in the sample.*
  9. Add 400 µL of Direct-zol^TM^ RNA PreWash buffer (part of Direct-zol^TM^ RNA Miniprep kit) to the column and centrifuge at 13,000 *× g* for 1 min. Discard the flow through and repeat this step one more time.
*Note: RNA PreWash buffer is provided as a concentrate buffer. You should dilute it with ethanol according to the manufacturer’s instructions.*
  10. Add 700 µL of RNA wash buffer to the column and centrifuge at 13,000 *× g* for 1 min to remove the wash buffer.
*Note: RNA wash buffer is provided as a concentrate buffer. You should dilute it with ethanol according to the manufacturer’s instructions.*
  11. Centrifuge the empty column present in a new collection tube at 13,000 *× g* for 5 min to ensure complete removal of the ethanol that is present in RNA PreWash buffer and RNA wash buffer.
    ***Note:*** *It is important to get rid of ethanol since any residual amount can impact the downstream qPCR steps and/or the yield and purity of extracted RNA.*
  12. Transfer the column carefully into an RNase-free tube, add 50 µL of DNase/RNase-free water directly to the column matrix, incubate at room temperature for 2–5 min, and then centrifuge at 13,000 *× g* for 1 min.
  13. Repeat step B16 using the RNA eluted from the column. Transfer the eluted RNA into the same column, incubate at room temperature for 2 min, and then centrifuge at 13,000 *× g* for 1 min.
    **Figure 2.**
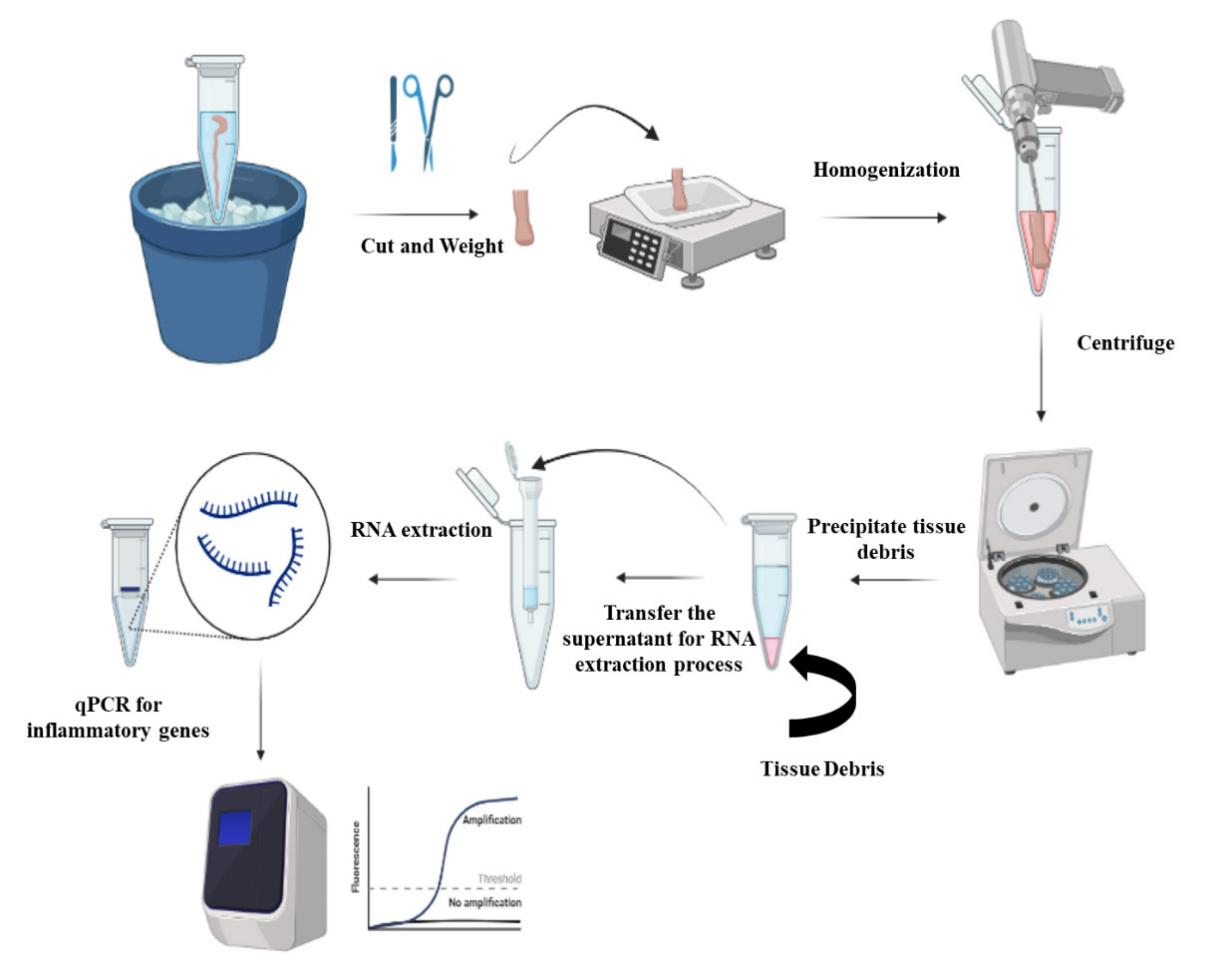
Steps of RNA isolation from the colon. Colon samples are cut into small pieces, weighted, and then homogenized. The homogenized samples are centrifuged to remove the tissue debris and the supernatants are processed for RNA extraction. The extracted RNA is used for quantification of the transcripts of inflammatory cytokines.
- **RT-qPCR for the inflammatory transcript in the mouse colon**
  **Conversion of RNA into cDNA**
  1. Convert the extracted RNA (approximately 500–750 ng) into cDNA using qScript cDNA SuperMix according to manufacturer’s instructions.
  2. To a 0.2 mL thin-walled PCR tube placed on ice, add the components as shown in **Recipe 2**. The volume of RNA added depends on the concentration. Total RNA added should be between 500 and 750 ng. The volume of free water added depends on the volume of RNA, according to **Recipe 2**.
*Note: When comparing samples, it is preferred to use the same amount of RNA. For example, to compare the transcripts from mouse colon derived from air-exposed groups and from JUUL mango pods, use the same amount of RNA to form cDNA; either 500 or 750 ng for all samples or in between concentrations.*
  3. Vortex the mixture gently and then centrifuge for 10 s to collect contents.
  4. Place the PCR tubes in a MiniAmp Plus thermal cycler using the following program (Table 1):
    **Table 1.** PCR program for synthesis of cDNA using qScript cDNA synthesis kit

Number of cycles	Temperature	Time
1	25 °C	5 min
1	42 °C	30 min
1	85 °C	5 min
	4 °C	Hold
  5. Dilute the cDNA product into 1/5 or 1/10 using nuclease-free water and store the diluted cDNA at -20 °C until analysis.
  **qRT-PCR reaction system**
  1. Use the synthesized cDNA as a template for qPCR reaction with 2× SYBR Green qPCR Master Mix.
  2. Using a MicroAmp^TM^ optical 96-well reaction plate, add the PCR reaction mixtures according to **Recipe 3**.
  3. Add optical adhesive covers GPLE to the plate, put in the machine, and run the following PCR program (Table 2):
    **Table 2.** PCR program

Step	Hold	PCR (40 cycles)			Melt curve (1 cycle)		
		**Denaturation**	**Anneal**	**Extend**			
Temperature	95.0 °C	95.0 °C	60.0 °C	72.0 °C	95.0 °C	60.0 °C	95.0 °C
Time	10 min	15 s	30 s	30 s	15 s	60 s	15 s

## Data analysis

High-yield purified RNA extraction is possible from mouse colon tissue. Using the RNAlater solution helps preserve tissues and/or the RNA for a longer period at -80 °C **(Figure 1)**. The previous step aids in the extraction of many samples at the same time to minimize the inter-assay procedure. It is important to weigh the colon piece undergoing the RNA extraction process **(Figure 2)**. Since each column has a maximum capacity, including too large samples could lead to blockage of the column, and therefore negatively impact all the downstream process of RNA extraction. The addition of TRI reagent stops RNase enzymes and neutralizes any infectious agents in the samples; therefore, it helps in improving the quality and stability of extracted RNA. Following the previously described procedure, you can assess the concentration and purity of RNA using a Nanodrop spectrophotometer. For the concentration, you may obtain ~100 ug of total RNA from the previous procedure. The purity of extracted RNA can be assessed by the measurement of absorbance (A_260/280 _and A_260/230_). The acceptable value for pure RNA in terms of A_260/280 is _1.8–2.2 or a value >1.8. A value lower than 1.8 indicates the presence of contaminants that absorb at 280 nm, such as protein and phenol. Similarly, 260/230 values >1.8 indicate pure RNA, while values lower than 1.8 indicate contamination with TRI reagent that absorbs at 230 nm.

For the qRT-PCR, the cycle threshold (Ct) value for the target gene was calculated using QuantStudio Design & Analysis Software, and then normalized to the housekeeping gene (∆CT). For comparison of the relative transcript expression between the different groups of mice, we used the formula ∆∆CT. For example, to compare the relative IL-6 transcript expression, we determined:

∆CT for IL-6 in JUUL exposed mice = CT _IL6_ - CT _housekeeping gene_)

∆CT for IL-6 in air-exposed mice = CT _IL6 _- CT _housekeeping gene_)

∆∆CT = ∆CT for IL-6 in JUUL exposed mice - ∆CT for IL-6 in air-exposed mice

Further data analysis for relative gene expression can be done using GraphPad Prism.

## Notes

1. **This method is suitable for RNA extraction from different mouse organs**
  Although the previous protocol focused mainly on the mouse colon, it can be applied to extract RNA from different mouse organs such as the spleen, liver, brain, lung, etc. We previously used this protocol to assess the inflammatory responses in the liver, spleen, cecum, and intestine of mice infected with *Salmonella* (Sayed et al., 2021). The liver and spleen are thicker than the intestine; therefore, the volume of TRI reagent should be increased to 800 μL and the homogenization time should be increased to 3–5 min.
2. **The quality of RNA and PCR products is important in the interpretation of the results**
  The quality of extracted RNA plays an important role in PCR reactions. The presence of impurities, which can be determined by the measurement of absorbance A_260/280 and _A_260/230_, could impact the downstream processing. Also, the primer design is important in the qPCR reaction. It is important to check the melting curve for each gene and confirm it is a single peak. The presence of several peaks indicates poor primer design and the possibility of primer dimer that affects the final interpretation of the results.

## Recipes

1. **DNase I treatment preparation**
  **Table:** 

Reagent	Volume
DNase I (1 U/µL)	5 µL
DNA digestion buffer	75 µL
  These components are parts of Direct-zol^TM^ RNA Miniprep kit. DNase I is provided as lyophilized powder. Reconstitute it using with DNase/RNase-Free Water. The volume of added water depends on the unit in DNase I powder to reach the final concentration of 1 U/µL. For example, add 250–275 µL water for 250 U of DNase I powder and 50–55 µL water for 50 U of DNase I powder. Mix well and then store the aliquots at -20 °C until use.
2. **Synthesis of cDNA from RNA using qScript cDNA synthesis Kit**
  **Table:** 

Reagent	Volume to add
qScript reaction mix (5×)	4 μL
RNA (0.5–0.75 µg)	Variable
Nuclease-free water	Variable
Total volume	20 μL
3. **qPCR reaction mixture**
  **Table:** 

Component	Volume per 10 μL reaction	Final concentration
2× SYBR Green qPCR Master Mix including ROX ^a^	5 μL	1×
cDNA (50–100 ng)^ b^	2 μL	10–20 ng ^b ^/ reaction (10 μL)
Forward primer (4 μM) ^c^	1 μL	0.4 μM
Reverse primer (4 μM) ^c^	1 μL	0.4 μM
Water	1 μL	
Total volume ^d^	10 μL
  ^a ^Add ROX reference dye 2 (low concentration) to the Green Master Mix according to the manufacturer's instructions, to reach a 1× final concentration.
  ^b ^The amount of cDNA formed in Recipe 2 is 500 ng, and dilution (1/5 or 1/10) is done to synthesized cDNA to become 50–100 ng. In general, 5-100 ng cDNA per reaction is acceptable.
  ^c^ For the sequences of primers used in the amplification of inflammatory transcripts in the colon please refer to Moshensky et al. (Moshensky et al., 2022)
  ^d ^Regarding the evaluation of the transcripts of inflammatory cytokines, 10 μL of reaction is sufficient since the abundance of these genes in the colon is high. For low-abundance genes, it is recommended to use a higher-volume reaction system (20–50 μL).
